# Compact Sub 6 GHz Slot Multiantenna System for 5G Laptops

**DOI:** 10.3390/mi14030626

**Published:** 2023-03-09

**Authors:** Shu-Chuan Chen, Chang-Sheng Wu, Shao-Hung Cheng, Chih-Chung Lin

**Affiliations:** Electrical and Electronic Engineering Department, Chung Cheng Institute of Technology, National Defense University, Taoyuan 335, Taiwan; scchen0319@gmail.com (S.-C.C.); johnson851122@gmail.com (C.-S.W.); tommylin98@gmail.com (C.-C.L.)

**Keywords:** laptop antennas, multi-input multi-output (MIMO) antennas, slot antennas, 5G antennas

## Abstract

A sub 6 GHz dual-band closed-slot multiantenna system for 5G laptops is proposed in this paper. It was installed in a clearance space, with dimensions og 217 × 3 mm^2^ and 1 mm away from the upper edge of the screen ground plane. The dimensions of the clearance space were the same as those of a multisystem consisting of six antennas. The dimensions of the single closed-slot antenna were 32 × 3 mm^2^ (0.368 λ × 0.034 λ, where λ equals the free-space wavelength of 3450 MHz. The antenna was coupled to an asymmetric T-shaped feed-in section equipped with a chip capacitor for exciting one-half and full wavelength resonance modes of the closed-slot to encompass sub 6 GHz 3300–3600 MHz and 4800–5000 MHz dual-band operations. The design of the antenna features a long and straight slot to generate the high-order mode of the closed slot in the high-frequency (4800–5000 MHz) band (not the low-frequency (3300–3600 MHz) multiplier band). Its structure is simple, and the width of its slot is only 3 mm. The antennas were arranged to be 5 mm apart in the same direction and in parallel to form a six-antenna system in order to utilize the weak electric fields located at the two closed ends of the closed-slot structure when the closed slot was excited. It showed excellent envelope correlation coefficients (ECCs) and isolation performance without the installation of isolation elements. The measured fractional bandwidth of the antenna was 10.15% and 6.73% at the center frequencies of 3450 MHz and 4900 MHz, respectively. Its measured isolation was always over 10 dB, and the efficiency was between 46% and 76%. The ECCs of the system calculated from the measured complex E-field radiation pattern were all below 0.2, which means that it is ideal for use in laptop devices with a high screen-to-body ratio and a metal back cover.

## 1. Introduction

In recent years, consumers have shown a preference for mobile communication products that offer high transmission rates, portability, lightweight, and large screen displays. Manufacturers have introduced a range of slim laptops with high screen-to-body ratios to meet these demands. However, the limited space in these devices has resulted in less space for antennas. As a result, the characteristics of many antennas installed in mobile communications devices are evolving into smaller sizes and lower profiles. Several multiple input, multiple output (MIMO) multiantenna systems for mobile devices and 5G applications [1,2,3,4,5,6,7,8,9,10,11,12,13,14,15,16,17,18,19,20,21,22,23,24,25,26,27,28] have been continuously proposed with the rapid spread of 5G MIMO technology, which also include the 5G MIMO multiantenna systems for mobile phones. In these systems, multiple antennas are configured at appropriate distances to maintain a certain degree of isolation. The spacing between the antennas in them is about 10–30 mm [2,3,4,5,6,7,8,9,10,11,12,13]. Some designs incorporate isolation elements to optimize the degree of isolation between antennas [14,15]. However, they occupy more of the limited space available in devices.

A certain distance between two antennas is necessary for achieving a good degree of isolation. There are several multiantenna systems configured in close distances, in which the degree of isolation between antennas is optimized with the application of a decoupling mechanism. For example, there are designs in which the branch points of two antennas are configured close to each other [16,17,18], designs in which a chip inductor is added to the end of two antennas’ branches [19,20], designs in which a chip capacitor is added to the common shorted branch of two antennas to form a band-pass resonant structure for the optimization the degree of isolation [21], and designs in which a band-pass resonant structure consisting of distributed capacitors and inductors is added to the shorted branch of two antennas to achieve decoupling [22]. A novel mushroom-inspired dual-band electronic bandgap structure was proposed to improve the isolation between closely packed antenna elements [23]. The ground part is located at the substrate’s bottom to enhance the isolation performance of the MIMO antenna that was designed [24]. The authors employed a decoupling inductor and stop-band filter to reduce the mutual coupling and antenna size [25]. This is the first instance where a decoupling inductor was shared by two bands. To reduce mutual coupling, two decoupling branches (DBs) were implemented [26]. The implementation of the DBs effectively reduces mutual coupling at 3.5 GHz, while having minimal impact on mutual coupling at 4.9 GHz. In [27], a single antenna consisted of an H-shaped radiating element fed to the main board of the smartphone. Each side edge consisted of four radiating elements, and the gap between each antenna element was optimized to achieve low mutual coupling. In addition, there is a two-antenna design with two different excitation mechanisms that is able to achieve good isolation without the need for a distance between the two antennas. The profile height of the two-antenna element is 4 mm, and its length is 30 mm. It can cover the 5G 3400–3800 MHz band [28].

The MIMO antenna systems use slot structures [6,7,8,9,10,11,12,13], which can be seamlessly integrated with metallic surroundings and are particularly suitable for devices with metal frames or back covers. Notably, refs. [10,11,12,13] belong to the state-of-the-art literature within the past year. In [10], the authors proposed eight identical small inverted F-shaped folded slots etched onto a metal frame as a highly isolated MIMO antenna. Effective mitigation of the coupling between antenna elements can be achieved through the ideal arrangement of the positions of eight antenna elements. In [11], the authors proposed an eight-element antenna system that operates at sub 6 GHz. The system utilizes a modified E-slot on the ground, effectively reducing the coupling between the different antenna components by suppressing the ground current effect. The MIMO antenna structures in [10,11] all adopt the open-slot design. Such design would require multiple openings in the exterior of a mobile device, which would detract from its esthetic appearance. Therefore, the ideal solution for mobile communication devices with metal back covers is to adopt a closed-slot structure without any openings. In [12], the authors proposed dual-band closely spaced closed-slot MIMO antennas for WiFi applications. The decoupling process involves simply etching slot lines based on the cancellation of the common and differential modes. In [13], the authors proposed a MIMO antenna structure that comes from the deployment of octagon-shaped resonant slots within the metallic ground plane. Due to the unique closed slot in the ground plane, wideband impedance was achieved. Although MIMO antenna structures with closed slots have been proposed in [12,13], exciting high resonant modes that are not frequency-doubling of low resonant modes using long and straight slot structures will pose a challenge.

To solve the limitations of the slotted and MIMO antennas, we designed a closed-slot dual-band 5G MIMO six-antenna design for laptops to be installed in 217 × 3 mm^2^ clearance space and at 1 mm from the top edge of the ground plane of screens. Its structure is simple. The width of its slot of only 3 mm. The first characteristic of this closed-slot antenna is that the high-frequency band (4800–5000 MHz) is not a multiplier of the low-frequency band (3300–3600 MHz). To support sub 6 GHz dual-band operations at 3300–3600 MHz and 4800–5000 MHz, an asymmetric T-shaped feed-in monopole with a 0.5 pF chip capacitor is used to excite the closed-slot antenna and trigger one-half and full wavelength resonance modes. The current distribution is varied due to a slight modification in the length of the central T-shaped stub [29]. The second characteristic of the proposed design is that the configuration distance between the two slots is only 5 mm, to form a six-antenna system in the same and parallel direction. By utilizing the weak electronic fields located at the two closed ends of the closed slot structure when it is excited, the antennas provide excellent envelope correlation coefficient (ECC) and isolation without the need for any decoupling elements. All the measured degrees of isolation were over 10 dB, and its efficiency was between 46% and 76%. The ECC values calculated from the measured complex electronic field radiation patterns were all below 0.2.

The organization of the paper is as follows: Section 2 presents the MIMO antenna system design details. Then, the radiation performance of the antenna is discussed in Section 3, including reflection coefficients, transmission coefficients, ECC, efficiency, and radiation pattern. The ergodic channel capacity and its parametric study are described in Section 4 and Section 5, respectively. Section 6 presents the MIMO antenna system characteristics’ analysis, before the conclusion is presented in Section 7.

## 2. Proposed MIMO Antenna System Design

**Figure 1:**
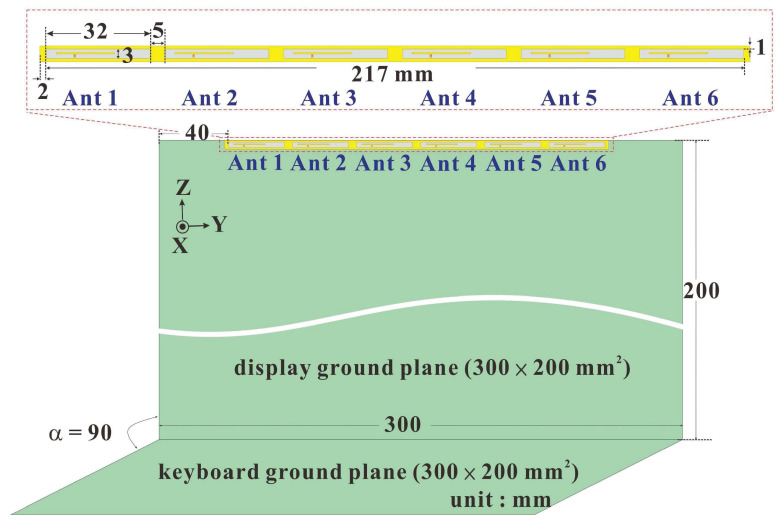
Overall design of the six 5G MIMO antennas.

The overall design of the dual-band 5G MIMO six-antenna system proposed in this paper is shown in Figure 1. The design is based on a screen ground plane and a keyboard ground plane with the dimensions of 300 × 200 mm^2^, which is approximated to the dimensions of a 13" laptop in the market today. Copper sheets were used to make the screen and keyboard ground planes. There was a clearance space of 217 × 3 mm^2^ at the position 1 mm from the upper edge and 40 mm from the left edge of the screen ground plane, which was intended for the installation of a 5G MIMO six-antenna system. The dimensions of the clearance space are the same as the ones of the six-antenna system. The dual-band 5G MIMO multiantenna system (including Ant 1, Ant 2, Ant 3, Ant 4, Ant 5, and Ant 6) was composed of six identically structured and sized closed-slot antennas configured 5 mm apart from each other, side by side, in the same direction. The dimensions of each antenna was 32 × 3 mm^2^. The six antennas were printed on a substrate made of FR4 glass fiber with a thickness of 0.4 mm. The dimensions of the substrate were 221 mm × 5 mm with a relative dielectric constant of 4.4 and a loss tangent of 0.02. The ground pads were printed around the FR4 glass fiber substrate and were connected to the screen ground plane. A ground pad was 2 mm long and 1 mm wide at the top and bottom. To maximize space utilization in laptops and enhance the operational bandwidth of antennas, a 300 × 3 mm^2^ copper sheet was vertically attached to the upper edge of the screen ground plane. This increased the bandwidth of the antennas, allowing each one to support sub 6 GHz dual-band operations at 3300–3600 MHz and 4800–5000 MHz.

**Figure 2:**
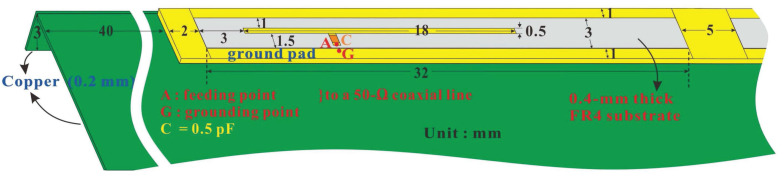
Detailed structure of a single 5G antenna (Ant 1).

Figure 2 depicts the detailed design for one of the 5G antennas. As the 5G six antennas were the same structure and size, Ant 1 is taken here as the example for the illustration of the structure of a single antenna. With a 32 × 3 mm^2^ rectangular closed-slot, a single antenna was coupled with an asymmetric T-shaped feed-in section with a 0.5 pF chip capacitor for exciting the one-half and full wavelength resonance modes of the closed slot to cover the sub 6 GHz dual-band operations at 3300–3600 MHz and 4800–5000 MHz. Chip capacitors were used here to improve the impedance matching in the low- and high-frequency bands to effectively trigger the low-frequency modes and to adjust the resonant frequencies of the high-frequency modes. In addition, T-shaped asymmetrical left and right branches were employed for adjusting the coupling levels at low and high frequencies. The signal feed-in point in the figure is denoted as point A, and it was connected to the inner conductor of a mini coaxial line with a characteristic impedance of 50 ohms. The outer conductor of the mini coaxial line was connected to point G, which was a ground short-circuit point on the metal ground plane of the screen.

The six-antenna system was designed with the concept of a closed-slot configuration, which could effectively excite the one-half wavelength and full wavelength resonance modes of the closed-slot structure at both low and high frequencies. When exciting the low- and high-frequency modes, the strength of the electric fields at the left and right ends of the slot decreased. As a result, the likelihood of coupling excitation affecting the neighboring slot antennas was reduced, resulting in a closed-slot antenna that can be closely integrated into a multiantenna system with excellent isolation and ECC performance.

## 3. Experiment and Measurement Results

**Figure 3:**
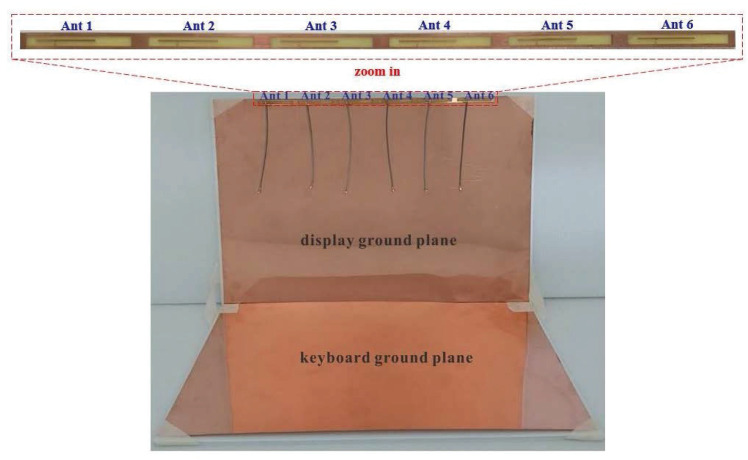
Overall configuration of the 5G MIMO six-antenna system.

**Figure 4:**
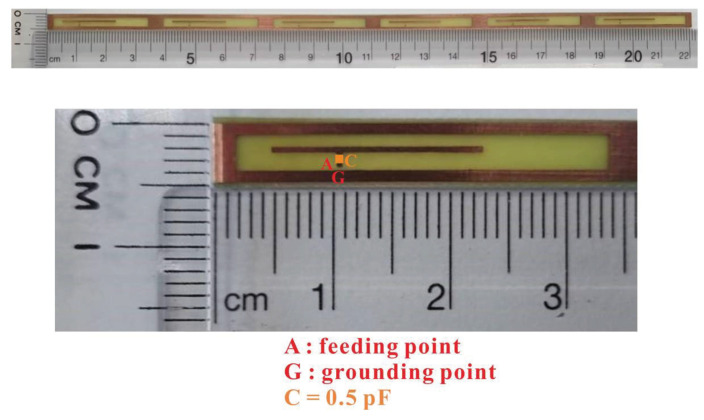
Single closed-slot antenna detail structure.

The overall design of the 5G MIMO six-antenna system is shown in Figure 3, and the details of the 5G MIMO six-antenna system and the single antenna are shown in Figure 4. Their measured dimensions are shown in Figure 1 and Figure 2. A simulation study using a commercially available tool, High Frequency Structure Simulator (HFSS), was conducted [30]. The reflection coefficients were set to below −6 dB (i.e., VSWR = 3:1). Based on the widely used design guidelines for mobile antennas, the transmission coefficients between these antennas were set to below −10 dB [2,3,4,5,6,7,8,9,14,15,16,17,18,19,20,21,22,25,26,27,28].

**Figure 5:**
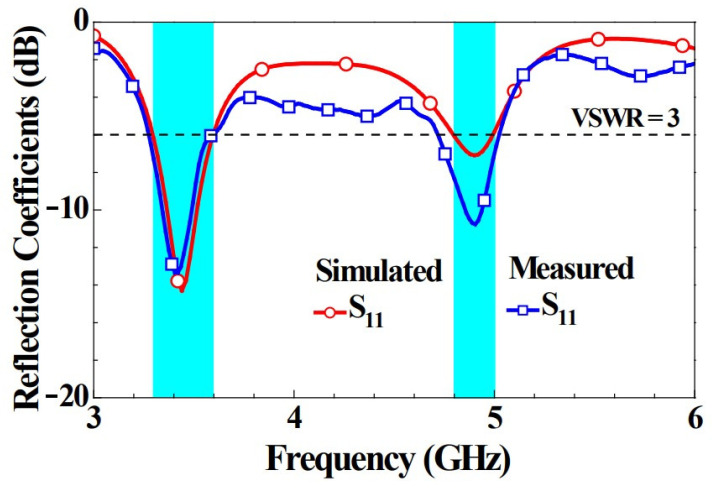
Simulated and measured reflection coefficients for Ant 1.

As antenna elements 2343 the same with only a slight difference in the configuration position for the six 5G MIMO antennas, the impedance matching was roughly the same. To keep the diagram from being complicated with many curves, only simulated S_11_ and measured S_11_ were chosen as the representative reflection coefficients in Figure 5. We can see that the unit element was able to generate two resonance modes to encompass the sub 6 GHz dual-band operations of 3300–3600 MHz and 4800–5000 MHz. The simulated and measured data were in good agreement, and the reflection coefficients observed were all above −6 dB in both the simulation and measurement. We noticed that the mismatch in the values of the reflection coefficients in both bands was mainly due to the presence or absence of the coaxial line. The coaxial line was not added in the simulation, but a 70 mm one was used as the feed in the implementation. The loss of the coaxial line caused slight differences in the simulated and measured reflection coefficients.

**Figure 6:**
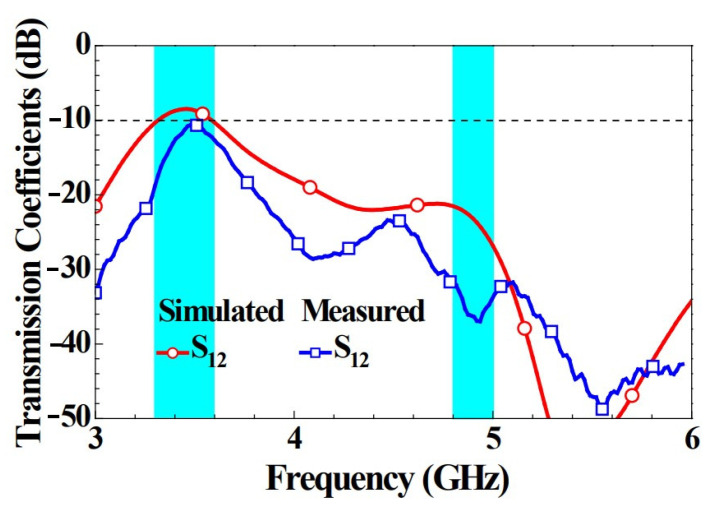
Simulated and measured transmission coefficients for Ant 1 and Ant 2.

S_12_ is sufficient to explain the isolation when antenna elements are symmetric. In the same way, only simulated S_12_ and measured S_12_ were chosen as the representative transmission coefficients for Figure 6. This was primarily in consideration of the fact that the transmission coefficients between the two adjacent antennas would be higher than those among the antennas with more space in between [6]. It is clearly indicated in the figure that the simulated transmission coefficient of the unit element in the sub 6 GHz bands of 3300–3600 MHz and 4800–5000 MHz was not more than −9 dB. However, the measured transmission coefficient was higher than −10 dB. The distance between the adjacent antennas among the six antennas was only 5 mm, and the degree of isolation was still better than 10 dB, which is excellent performance compared with that of other devices with multiple antennas [6,7,16]. The efficiency of the antennas should be more than 40% for application in practical scenarios [6].

**Figure 7:**
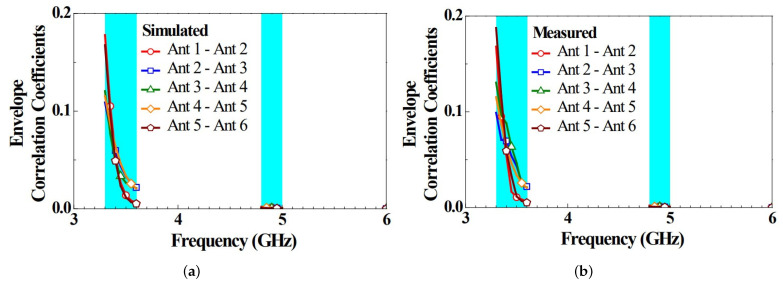
ECCs calculated from the complex E-field radiation patterns (**a**) simulated and (**b**) measured for the 5G antenna system.

In addition to the degree of isolation between the antennas, the ECC serves as an essential reference parameter for MIMO antenna systems. Generally, an ECC of below 0.5 meets the criterion for practical applications [31]. The ECCs calculated from the simulated and measured complex electronic field radiation patterns for the dual-band 5G MIMO six-antenna system are shown in Figure 7, where the ECCs are all below 0.2 in the bands. This confirms the good channel independence among the antennas of this 5G MIMO six-antenna system. The simulations and measurements presented above exhibited several discrepancies, which could be attributed to the absence of transmission lines in the simulations. It might also have been caused by the parameters of either soldered or FR4 substrates or copper sheets, which might be slightly different from the simulations in practical application cases.

**Figure 8:**
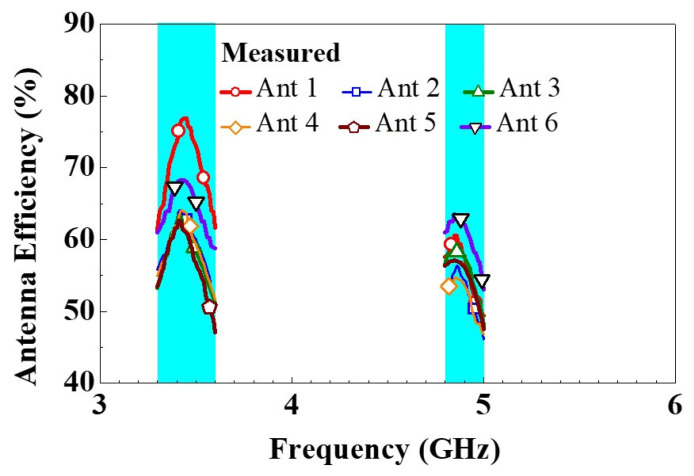
Antenna efficiencies measured for the 5G antenna system.

The measurement efficiency of the 5G MIMO six-antenna system is shown in Figure 8, and it ranged from 47% to 76% in the low-frequency band and 46% to 60% in the high-frequency band. Additionally, we found that Ant 1 and Ant 6 were significantly more efficient than the other antennas in the low-frequency band primarily due to their positions. Both the antennas had one side not equipped with other antennas and were therefore less affected by the other antennas. The other side that was not fitted with antennas also had more space of grounded metal, which helped to more evenly distribute the currents around the antennas. These are the reasons for the better efficiency of Ant 1 and Ant 6 compared with that of the other antennas.

**Figure 9:**
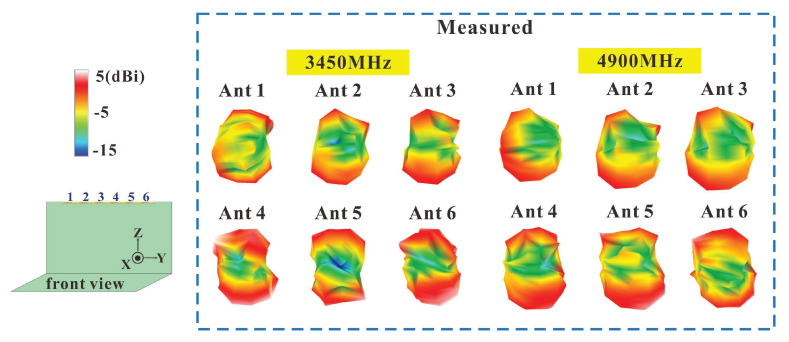
Measured 3D radiation patterns for six antennas at 3450 MHz and 4900 MHz.

The measured 3D radiation patterns of the six-antenna systems operating at 3450 MHz and 4900 MHz are provided in Figure 9. The radiation patterns indicate that the far-field radiation patterns of the six antennas were similar but not identical, making the far-field radiation patterns of this design more independent. This also echoes the finding that the ECCs of the six-antenna systems were all below 0.2 in the bands of operation. This six-antenna design is therefore well suited for the operation of MIMO systems. The antenna patterns, which are similar but not identical, can be primarily attributed to the small differences in the positions of the antennas on the ground plane of the screen.

## 4. Channel Capacity of a Six-Antenna Array

The ergodic channel capacity can be evaluated by averaging the capacities observed in a multipath Rayleigh fading environment. When a transmitter does not have channel state information (CSI), and its power is equally divided by each transmit antenna unit, its ergodic channel capacity can be calculated with the following formula [2]:

(1) $$\begin{array}{c} C=E\left\{\log_{2}\left[\det\left(I_{n_{r}}+\frac{\Gamma }{n_{t}}HH^{†}\right)\right]\right\}, \end{array}$$

where Γ is the signal-to-noise ratio (SNR), *H* is the wireless channel matrix, ()† is the Hermitian transpose of the matrix, $n_{t}$ is the number of transmit antennas, $n_{r}$ is the number of receive antennas, $I_{n_{r}}$ is the identity matrix of size $n_{r}$, det is the determinant, and *E* is the expectation concerning different channel realizations. In addition, the wireless channel matrix *H* in (1) is evaluated as follows [32]:

(2) H=Υ1122Hiid,

where $H_{iid}$ is the independently and identically distributed (i.i.d.) wireless channel matrix in a Rayleigh fading environment. Considering the effects of antennas in the test, the receiver correlation matrix Υ can be calculated by the following formula:

(3) Υ=Φ1122Υ¯Φ1122.

In (3), Φ is the diagonal efficiency matrix, i.e.,

(4) $$\begin{array}{cc} \Phi =\mathrm{diag} & \left[\eta_{1},\eta_{2},\cdots ,\eta_{n_{r}}\right], \end{array}$$

where $\eta_{i}$ is the efficiency of antenna *i*. The complex correlation coefficient matrix $\bar{\Upsilon }$ is expressed as [33]:

(5) $$\begin{array}{c} \bar{\Upsilon }=\left[\begin{array}{cccc} 1 & \rho_{12} & \cdots & \rho_{1n_{r}}\\ \rho_{12}^{*} & 1 & \cdots & \rho_{2n_{r}}\\ \vdots & \vdots & \ddots & \vdots \\ \rho_{1n_{r}}^{*} & \rho_{2n_{r}}^{*} & \cdots & 1 \end{array}\right], \end{array}$$

where ∗ is the conjugate operator, and $\rho_{ij}$ is the complex correlation coefficient between antennas *i* and *j*. The complex correlation coefficient can be expressed in terms of the S parameters of an antenna system as follows [34]:

(6) $$\begin{array}{c} \rho_{ij}=\frac{-(S_{ii}^{*}S_{ij}+S_{ji}^{*}S_{jj})}{\sqrt{\left(1-{\left|S_{ii}\right|}^{2}-{\left|S_{ji}\right|}^{2}\right)\left(1-{\left|S_{jj}\right|}^{2}-{\left|S_{ij}\right|}^{2}\right)}}, \end{array}$$

where $\left|\cdot \right|$ is the magnitude operator.

**Figure 10:**
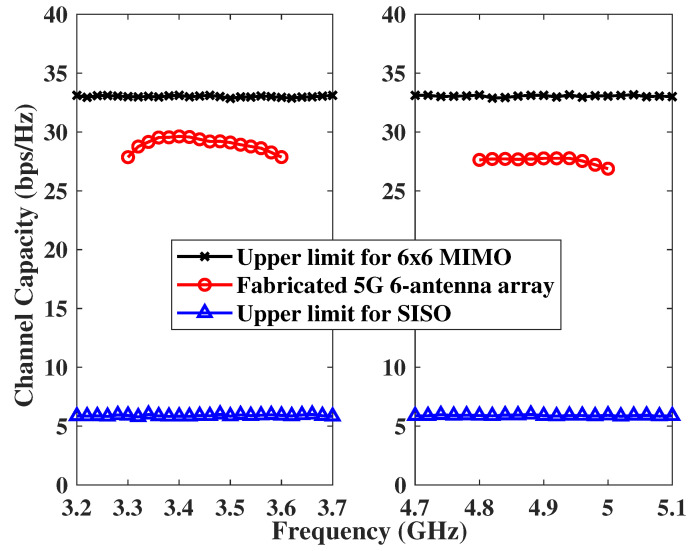
Calculated ergodic channel capacities of 5G antenna array prototype in 6 × 6 MIMO system.

The results of the calculated ergodic capacity with a 20-dB SNR are shown in Figure 10. The proposed 5G six-antenna array in free space was compared with the case of a six-antenna ideal 6 × 6 MIMO system and the case of one ideal single-input, single-output (SISO) system. The ideal transmitting and receiving antennas have 100% efficiency, null ECC, and i.i.d. channels with a Rayleigh fading environment. At each frequency point, all results of capacity were calculated by averaging 1000 channel realizations to ensure that they converged to specific values. In the free space, the capacity in 3300–3600 MHz was between 27.9 and 29.6 bit/s/Hz, while the capacity in 4800–5000 MHz was from 26.8 to 27.8 bit/s/Hz. The highest capacity value in the band of 3300–3600 MHz was 3 bit/s/Hz lower than the capacity of 6 × 6 MIMO i.i.d. channels. The proposed six-antenna array achieved capacity much larger than that of a SISO system; therefore, it is promising for future 5G laptop application.

## 5. Parametric Study

**Figure 11:**
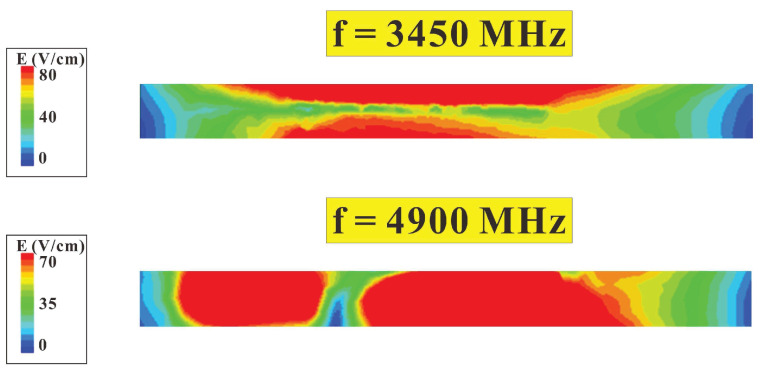
Electronic field simulation and distribution for single antennas at 3450 MHz and 4900 MHz.

To verify the operating principle of the antennas and to provide a clearer picture of the contribution path of each mode in different frequency bands, we analyzed the electronic field distribution of the antennas at the two resonant frequency points, 3450 MHz and 4900 MHz, in the low- and high-frequency bands. The simulated electronic field distribution of the single antenna at 3450 MHz and 4900 MHz is demonstrated in Figure 11. It is evident from the figure that at 3450 MHz operation, the electronic field distribution followed the one-half wavelength resonance of a closed slot, with the maximum field occurring at the center of the slot. At 4900 MHz operation, the electronic field distribution followed the full wavelength resonance of a closed slot, with two peak fields occurring on both sides of the slot. It is notable that the distribution of the two peak fields on both sides is slightly asymmetrical because of the T-shaped feed-in section with a long coupling interval adjacent to the left side.

**Figure 12:**
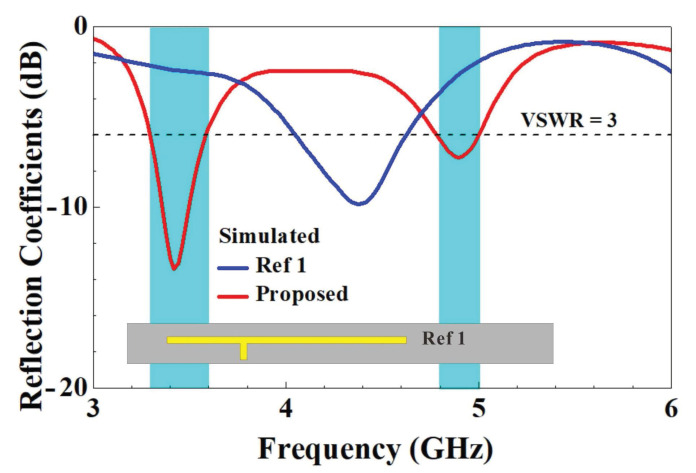
Comparison of the simulated reflection coefficient of a T-shaped feed-in section with and without a chip capacitance *C*.

In Figure 12, the simulated reflection coefficients of the proposed single-antenna T-shaped feed-in section with and without a chip capacitor are compared. The figure shows that the closed-slot antenna, featuring a basic T-shaped feed-in section, displayed a resonance mode at 4200 MHz. Notably, the real impedance of the antenna in the low-frequency band ranged between 25 and 125 ohms. However, its virtual impedance was too high to achieve a match. By adding a chip capacitor to the T-shaped feed-in section, the extremely high virtual impedance at low frequencies could be reduced to achieve a match, resulting in a resonance mode at 3450 MHz. At this point, the resonance mode at 4200 MHz also dropped as a result of the virtual impedance, shifting the resonance frequency to 4900 MHz in the sub 6 GHz high-frequency band and allowing the proposed single antenna to cover both the 3300–3600 MHz and 4800–5000 MHz dual-band operations required for sub 6 GHz antennas.

The design of the single antenna is characterized by the use of a long T-shaped metal wire segment for coupling and exciting the closed-slot configuration, which allows it to provide resonance modes at high frequencies. At low frequencies, the amount of coupling is too much for the virtual impedance that is too high to yield resonance modes. As a result, a suitable capacitance element was incorporated in the feed-in section for lowering the initially higher virtual impedance at low frequencies to achieve a match and generate a low-frequency resonance mode, and the original high-frequency mode was also boosted to 4900 MHz. As such, the asymmetric T-shaped feed-in section with a chip capacitor could effectively excite one-half and full wavelength resonance modes of the closed slot structure to encompass the 3300–3600 MHz and 4800–5000 MHz dual-band operations required for sub 6 GHz antennas.

In order to further understand the design parameters of the unit element slotted dual-band antennas, we analyzed five design parameters that affect the characteristics of antennas. The five design parameters included capacitance value *C*, the length *d* of the slotted hole, the coupling gap between the T-shaped feed-in monopole and the slotted hole, the length *r*’ of the right branch, and the length *t*’ of the right branch in the T-shaped feed-in monopole. The variation in parameter *C* affects both resonant modes. With the increase in *C*, two resonant points move to low frequency due to the virtual impedance transform. The optimized value of parameter *C* is 0.5 pF. A little resonant frequency offset of the first resonant mode arises with the increase in parameter *r*, and the major impact is caused for the second resonant mode. The final value of parameter *r* is 12 mm. With increases in parameter *t*, the impedance matching condition at the second resonant mode deteriorates and slightly shifts to high frequency. The ultimately optimized parameter *t* is 5.5 mm. As parameter *d* increases, the first resonant frequency significantly decreases, and the second resonant frequency shifts less. The final value of parameter *d* is 32 mm. As the parameter gap grows, the impedance matching condition for the first resonant mode improves and shifts toward higher frequencies. The ultimately optimized parameter gap is 1 mm. The parametric analysis of each design parameter is described below to understand the impact on the antenna characteristics.

**Figure 13:**
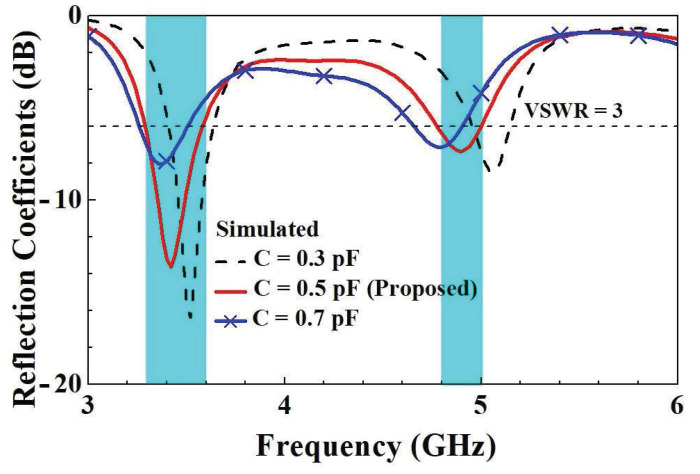
Comparison of simulated reflection coefficients with changes in *C*.

The comparison of the simulated reflection coefficient with a change in the capacitance value *C* is shown in Figure 13. It can be seen that when the capacitance value rose, there was a tendency for both low- and high-frequency modes to drop in frequency. This occurred mainly because when the capacitance value grows, the virtual impedance increases, causing the frequency to drop through zero virtual impedance. The resonance frequency points can therefore be adjusted by changing the capacitance value to modify the virtual impedance of the low- and high-frequency modes.

**Figure 14:**
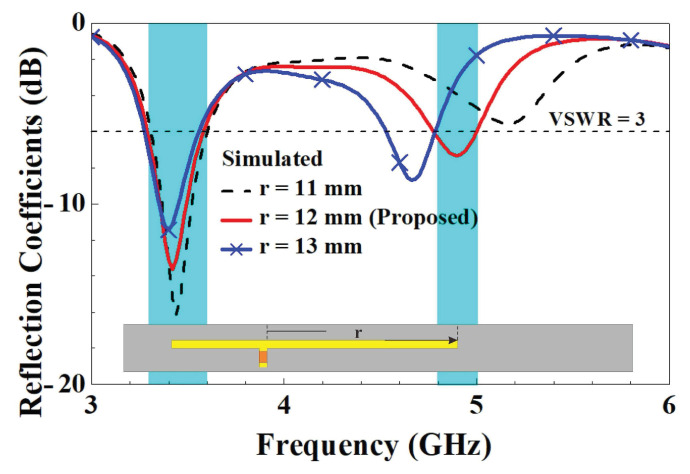
Comparison of simulated reflection coefficients with changes in parameter *r*.

**Figure 15:**
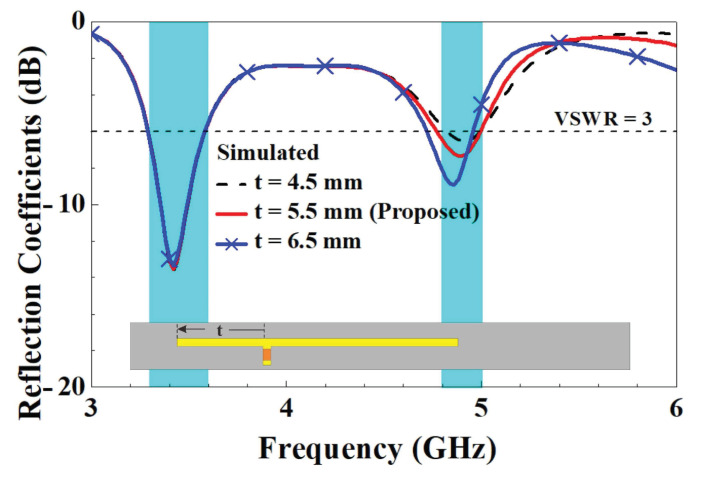
Comparison of simulated reflection coefficients with changes in parameter *t*.

Regarding the changes in the length of the left and right branches in the T-shaped feed-in section, Figure 14 compares the reflection coefficients for the right-side branch parameter *r*. The figure demonstrates that increasing the length of the right-side branch from 11 mm to 13 mm resulted in changes to the reflection coefficients. There was a clear tendency for both low- and high-frequency modes to decrease in frequency. This primarily occurred in the length where the right-side branch was situated in the distribution area of the strong electronic field when the low- and high-frequency modes were excited, and then the variations in length and the coupling levels had a marked effect on both low- and high-frequency modes. A comparison of the reflection coefficients for the left-side branch parameter *t* in the T-shaped feed-in section is provided in Figure 15, which indicates that when the left-side branch grew from 4.5 mm to 6.5 mm in length, there was a clear tendency for the high-frequency modes to fall in frequency. However, there was no significant change in the low-frequency modes. This was primarily in the length in which the left-side branch was situated in the distribution area of the strong/weak electronic fields when the high-/low-frequency modes were excited. The variations in length and the coupling levels thus had/did not have a marked effect on the high-/low-frequency modes.

**Figure 16:**
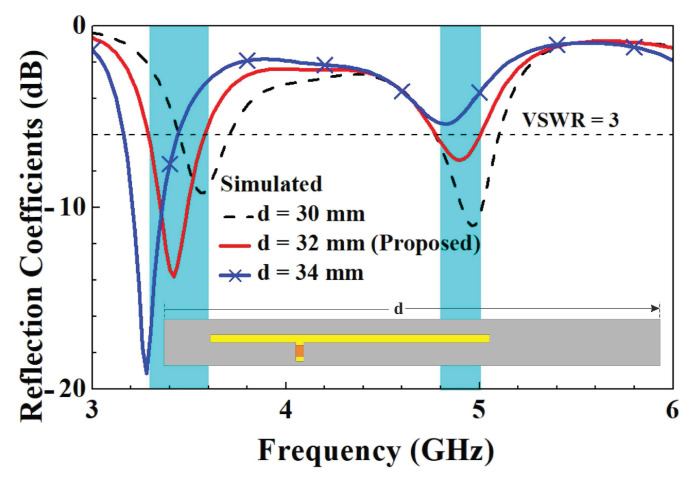
Comparison of simulated reflection coefficients with changes in parameter *d*.

**Figure 17:**
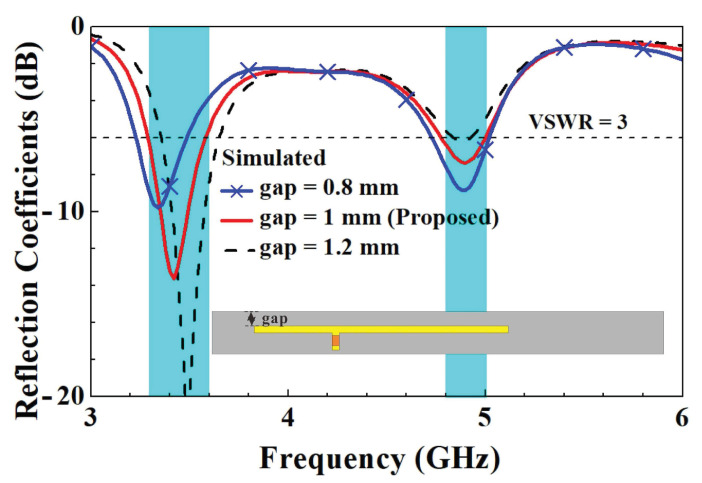
Comparison of simulated reflection coefficients with changes in parameter gap.

Then, a parameter analysis of the length *d* of the slotted hole was conducted. A comparison of the reflection coefficients for parameter *d* is shown in Figure 16. We can clearly see from the diagram that when the slot length was reduced from 34 mm to 30 mm, there was upward trends in both the low- and high-frequency modes. This was followed by an analysis of the variation in the coupling gap between the T-shaped feed-in section and the slotted hole. A comparison of the reflection coefficients for the parameter gap is shown in Figure 17. When the gap width grew from 0.8 mm to 1.2 mm, the coupling level between the low and high frequencies fell, resulting in a boost in the low-frequency mode. The boost in the high frequencies was not significant, but the matching noticeably changed. Based on the analysis mentioned above, we could adapt the low- and high-frequency resonance frequencies and their matching by applying the capacitance value of the T-shaped feed-in section, the length of the left and right branches, and the coupling distance between them and the slotted holes. Using the aforementioned analysis, we could adjust the low- and high-frequency resonance frequencies and their matching by modifying various parameters. These parameters included the capacitance value of the T-shaped feed-in section, the length of the left and right branches, the length of the slotted hole, and the coupling distance between them.

## 6. MIMO Antenna System Characteristics and Advantages Analysis

**Figure 18:**
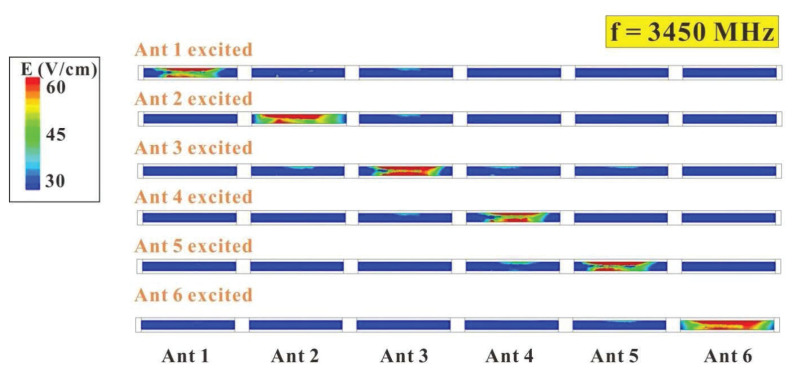
Simulated electronic field distribution of the six-antenna system at 3450 MHz.

**Figure 19:**
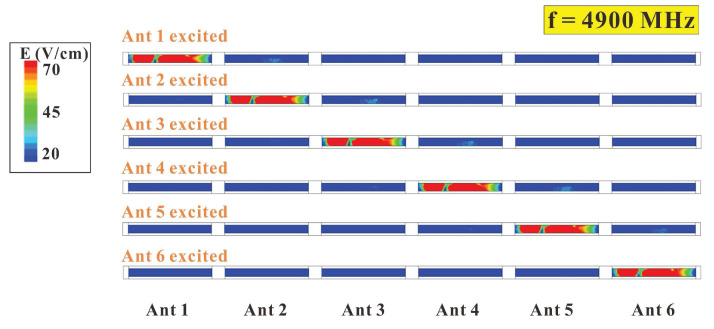
Simulated electronic field distribution of the six-antenna system at 4900 MHz.

To further investigate the degree of isolation of the six-antenna system, the electronic field distribution of the six-antenna system was also analyzed. The simulated electronic field distributions of the six-antenna system at 3450 MHz and 4900 MHz are shown in Figure 18 and Figure 19, respectively. It can be clearly seen in the diagrams that when the six-antenna system was operated at 3450 MHz and 4900 MHz, no matter which antenna was excited, the other antennas did not exhibit a significantly excited electronic field distribution. The same situation occurred at 4900 MHz. This echoes the good degree of isolation and ECC performance of this six-antenna system, which may be associated with the structural design of these 5G antennas.

To highlight the advantages of our proposed MIMO antennas, we provide a comparison of MIMO antennas reported in the literature in Table 1. The table presents a comparison of the dimensions, spacing between two-antenna units (in terms of the wavelength λ at the center frequency for the first resonant mode [35]), isolation, and various radiation performance for 5G sub 6 GHz dual-band frequencies. The table shows that our proposed MIMO antennas are more compact than those reported in the literature with dual-band frequencies.

## 7. Conclusions

This paper presented a closed-slot sub 6 GHz dual-band MIMO six-antenna system for 5G laptops with metal back covers. This six-antenna system is arranged with long and straight closed slots that have the same structure and size in a continuous and compact manner, which not only has a simple structure but can also meet the requirements of a large screen with a high screen-to-body ratio. Employing an asymmetric T-shaped feed-in section with a chip capacitor, the closed slots can be excited to produce half-wavelength resonant modes that cover the 3300–3600 MHz range required for the sub 6 GHz low-frequency band. Additionally, full-wavelength resonant modes can be excited to cover the 4800–5000 MHz range required for the sub 6 GHz high-frequency band that is not part of the sub 6 GHz low-frequency band’s octave range. The six-antenna system can be set up in a compact, continuous manner in 5 mm intervals without requiring any isolation elements, which not only achieves good space utilization, multiple antenna efficiency, ECC and isolation, but also has a beautiful metal look.

## Figures and Tables

**Table 1:** Performance comparison of MIMO antenna systems.

Ref.	Single Antenna Size (Width × Length) (mm)	Operating Bands (MHz)	Spacing between Two-Antenna Units (mm)	Isolation (dB)	Eff.	ECC	Antenna Structure	More than Six-Antenna System
[10]	3.8 × 6.78 (0.06 λ × 0.11 λ @ 4.70 GHz)	4400–5000	≥ 31 (0.49 λ @ 4.70 GHz)	≥22	≥50%	≤0.049	opened-slot	achieved
[11]	5.6 × 17.8 (0.07 λ × 0.21 λ @ 3.53 GHz)	3400–3650	≥19.7 (0.23 λ @ 3.53 GHz)	≥21	≥34%	≤0.01	opened-slot	achieved
[12]	3 × 37 (0.02 λ × 0.30 λ @ 2.45 GHz)	2400–2500 5200–5450	≥10 (0.08 λ @ 2.45 GHz)	≥15	≥60%	≤0.08	closed-slot	N/A
[13]	20 × 20 (0.24 λ × 0.24 λ @ 3.58 GHz)	3350–3800	≥35 (0.42 λ @ 3.580 GHz)	≥15	≥90%	≤0.01	closed-slot	achieved
[16]	1 × 16 × 4.2 (0.01 λ × 0.18 λ × 0.05 λ @ 3.45 GHz)	3300–6000	only one two-antenna unit	≥10	≥56%	≤0.1	inverted-F	N/A
[19]	4 × 9.5 (0.05 λ × 0.11 λ @ 3.60 GHz)	3400–3800	only one two-antenna unit	≥17.5	≥50%	≤0.05	inverted-F	N/A
[23]	12.32 × 25.2 (0.13 λ × 0.27 λ @ 3.20 GHz)	2800–3600 4700–5600	only one two-antenna unit	≥35	N/A	≤0.02	patch	N/A
[24]	50 × 50 × 1.6 (0.43 λ × 0.43 λ × 0.01 λ @ 2.58 GHz)	2250–2900 5050–6025	only one two-antenna unit	≥19.3	≥60%	≤0.03	patch	N/A
[25]	4 × 9 (0.05 λ × 0.11 λ @ 3.50 GHz)	3400–3600 4800–5000	only one two-antenna unit	≥12	≥38%	N/A	monopole	achieved
[26]	3.5 × 9 × 5 (0.05 λ × 0.11 λ × 0.06 λ @ 3.60 GHz)	3380–3820 4750–5130	≥21 (0.25 λ @ 3.60 GHz)	≥14.5	≥ 60%	≤0.08	inverted-F	achieved
[27]	4 × 9 (0.05 λ × 0.11 λ @ 3.50 GHz)	3600–3900 5500–6300	≥22 (0.26 λ @ 3.50 GHz)	≥12	≥42%	≤0.035	monopole	achieved
this work	3 × 32 (0.03 λ × 0.37 λ @ 3.45 GHz)	3300–3600 4800–5000	5 (0.06 λ @ 3.45 GHz)	≥10	≥47%	≤0.2	closeed-slot	achieved

## Data Availability

Not applicable.
